# Systematic study of tissue factor expression in solid tumors

**DOI:** 10.1002/cnr2.1699

**Published:** 2022-08-13

**Authors:** Johann S. de Bono, Jeffrey R. Harris, Saskia M. Burm, Adriaan Vanderstichele, Mischa A. Houtkamp, Saida Aarass, Ruth Riisnaes, Ines Figueiredo, Daniel Nava Rodrigues, Rossitza Christova, Siel Olbrecht, Hans W. M. Niessen, Sigrid R. Ruuls, Danita H. Schuurhuis, Jeroen J. Lammerts van Bueren, Esther C. W. Breij, Ignace Vergote

**Affiliations:** ^1^ The Institute of Cancer Research Royal Cancer Hospital London UK; ^2^ Genmab Plainsboro New Jersey USA; ^3^ Genmab Utrecht The Netherlands; ^4^ Department of Gynaecology and Obstetrics, Division of Gynaecologic Oncology University Hospitals Leuven, Leuven Cancer Institute Leuven Belgium; ^5^ Department of Pathology VU Medical Center Amsterdam The Netherlands; ^6^ Lava Therapeutics Utrecht The Netherlands; ^7^ Merus Utrecht The Netherlands

**Keywords:** biopsies expression, immunohistochemistry, solid tumor, targeted therapy, tissue factor

## Abstract

**Background:**

Elevated tissue factor (TF) expression, although restricted in normal tissue, has been reported in multiple solid cancers, and expression has been associated with poor prognosis. This manuscript compares TF expression across various solid tumor types via immunohistochemistry in a single study, which has not been performed previously.

**Aims:**

To increase insight in the prevalence and cellular localization of TF expression across solid cancer types, we performed a detailed and systematic analysis of TF expression in tumor tissue obtained from patients with ovarian, esophageal, bladder, cervical, endometrial, pancreatic, prostate, colon, breast, non‐small cell lung cancer (NSCLC), head and neck squamous cell carcinoma (HNSCC), and glioblastoma. The spatial and temporal variation of TF expression was analyzed over time and upon disease progression in patient‐matched biopsies taken at different timepoints. In addition, TF expression in patient‐matched primary tumor and metastatic lesions was also analyzed.

**Methods and Results:**

TF expression was detected via immunohistochemistry (IHC) using a validated TF‐specific antibody. TF was expressed in all cancer types tested, with highest prevalence in pancreatic cancer, cervical cancer, colon cancer, glioblastoma, HNSCC, and NSCLC, and lowest in breast cancer. Staining was predominantly membranous in pancreatic, cervical, and HNSCC, and cytoplasmic in glioblastoma and bladder cancer. In general, expression was consistent between biopsies obtained from the same patient over time, although variability was observed for individual patients. NSCLC biopsies of primary tumor and matched lymph node metastases showed no clear difference in TF expression overall, although individual patient changes were observed.

**Conclusion:**

This study shows that TF is expressed across a broad range of solid cancer types, and expression is present upon tumor dissemination and over the course of treatment.

## 1 INTRODUCTION

Tissue factor (TF, F3, coagulation factor III, thromboplastin, or CD142) is a transmembrane glycoprotein that is the main physiologic initiator of coagulation when exposed to blood after injury.^1^, ^2^ TF also aids in wound healing by stimulating angiogenesis via the protease‐activated receptor‐2 (PAR2, F2RL1)‐mediated intracellular signaling pathway and hampers apoptosis via the Janus kinase (JAK)/signal transducer and activator of transcription (STAT) pathway.^3^ TF plays a critical role in embryonic development.^4^ Expression of TF under physiologic conditions is limited to some tissues,^5^ is predominantly found perivascular, and has been shown to be anatomically sequestered from blood.^5^, ^6^

Aberrant expression of TF in cancer was reported over four decades ago,^7^ and has been described in a wide range of solid tumors, including breast, ovarian, prostate, pancreatic, bladder, cervical, esophageal, and colon cancer, HNSCC, NSCLC, and glioblastoma compared to normal tissue^8^ (Table 1). TF expression in cancer is hypothesized to be induced by several mechanisms including loss of tumor suppressor genes (phosphatase and tensin homolog [PTEN] or p53), activation of oncogenes (e.g., K‐RAS and epidermal growth factor receptor variant III [EGFRvIII]), hypoxic tumor microenvironment, or transforming growth factor β [TGFβ] signaling.^9^ Mutations in, or amplification of the TF gene itself have not been described. In cancer, TF is thought to facilitate primary tumor growth, neo‐angiogenesis, tumor invasion, and metastasis,^10^ and TF expression consequently has been associated with poor prognosis in multiple solid cancers.^9^, ^11^, ^12^, ^13^ Expression across solid cancers makes TF an interesting therapeutic cancer target, and preclinical proof‐of‐concept studies have been described for TF‐targeting antibodies, antibody‐drug conjugates, immune‐conjugates, micro‐RNAs, and TF pathway inhibitors.^9^, ^14^, ^15^

**TABLE 1:** TF expression in solid cancer

Cancer type	Present study						Reported in literature	
	TF prevalence (%)^a^	TF intensity ≥2 (%)^b^	TF H‐score Median (Q1, Q3)		TF H‐score Mean ± SEM		TF prevalence (%)	
	*Combined Cytoplasm* + *Membrane*		*Cytoplasm*	*Membrane*	*Cytoplasm*	*Membrane*	*Any staining reported*	Reference (as per Suppl. reference list)
Glioblastoma	93% (56 of 60)	65% (39 of 60)	128 (70, 167)	5 (0, 15)	123 ± 9	15 ± 3	90%–95%	1–4
Pancreatic cancer	86% (51 of 59)	83% (49 of 59)	87 (41, 150)	102 (30, 175)	96 ± 9	103 ± 11	61%–100%	5–9
Cervical cancer	77% (47 of 61)	66% (40 of 61)	25 (3, 103)	80 (11, 181)	54 ± 8	108 ± 12	94%–100%	10–12
NSCLC	77% (46 of 60)	47% (28 of 60)	24 (4, 78)	23 (1, 70)	43 ± 6	45 ± 7	34%–88%	13–17
HNSCC	75% (45 of 60)	68% (41 of 60)	37 (6, 106)	77 (13, 149)	56 ± 7	86 ± 9.7	63%–100%	18, 19
Endometrial cancer	70% (42 of 60)	57% (34 of 60)	12 (3, 68)	44 (6, 104)	39 ± 6	62 ± 9	14%–100%	20, 21
Prostate cancer	68% (41 of 60)	52% (31 of 60)	30 (1, 94)	25 (1, 128)	55 ± 8	65 ± 10	47%–78%	22–26
Esophageal cancer	63% (39 of 62)	45% (28 of 62)	3 (0, 20)	27 (3, 120)	21 ± 5	63 ± 10	43%–91%	27, 28
Ovarian cancer	47% (28 of 60)	33% (20 of 60)	5 (1, 50)	5 (0, 50)	29 ± 6	28 ± 6	75%–100%	29–32
Bladder cancer	38% (22 of 58)	26% (15 of 58)	3 (0, 31)	0 (0, 4)	24 ± 5	9 ± 3	78%	33
TNBC	35% (16 of 46)	9% (4 of 46)	2 (0, 1)	1 (0, 11)	13 ± 4	10 ± 3	—	
Breast	10% (4 of 40)	8% (3 of 40)	0 (0, 1)	0 (0, 0)	4 ± 2	5 ± 3	31%–100%	34–41
Colon cancer	76% (22 of 29)	59% (17 of 29)	40 (4, 100)	53 (10, 133)	63 ± 13	84 ± 16	41%–100%	42–45

Abbreviations: HNSCC, head and neck squamous cell carcinoma; NSCLC, non‐small cell lung cancer; SEM, standard error of the mean; TNBC, triple‐negative breast cancer.

^a^ Percentage of cases with TF expression in at least 10% of tumor cells, intensity levels 1+, 2+, and 3+.

^b^ Percentage of cases expressing medium‐high (intensity 2+ or 3+) in at least 10% of tumor cells.

Comparison of reported TF expression levels between malignancies is difficult, because methodologies used to assess TF expression across individual studies are highly variable, with most studies focused on a single cancer type. In addition, little is known about the cellular localization of TF within tumor cells, the expression pattern in matched primary tumors versus metastatic lesions, or the dynamics of TF expression during disease progression.

In the present study, we set out to determine the prevalence, cellular localization, and the spatial and temporal expression patterns of TF in a broad panel of solid tumor biopsies, taken at various time points and stages of disease, and from primary and metastatic lesions, using validated reagents and IHC methods.

## 2 MATERIALS AND METHODS

### 2.1 Tumor cell lines and tissue samples

#### 2.1.1 Tumor cell lines

Human PC3 (prostate carcinoma, ATCC, Cat# CRL‐1435, RRID:CVCL_B0CN) DU145 (prostate carcinoma, ATCC Cat# HTB‐81, RRID:CVCL_0105), LNCaP (prostate carcinoma, ATCC Cat# CRL‐1740, RRID:CVCL_1379), 786‐O (renal clear cell adenocarcinoma, ATCC Cat# CRL‐1932, RRID:CVCL_1051), HCT 116 (colorectal carcinoma, ATCC Cat# CCL‐247, RRID:CVCL_0291) and BxPC‐3 (pancreas carcinoma, ATCCD Cat# CRL‐1687, RRID:CVCL_0186) cells were obtained from the American Type Culture Collection (ATCC; Manassas, VA, USA). The epidermoid adenocarcinoma cell line A431 (DSMZ Cat# ACC 91, RRID:CVCL_0037) was obtained from the Deutsche Sammlung von Mikroorganismen und Zellkulturen GmbH (DSMZ, Braunschweig, Germany), LNCaP95 (prostate carcinoma, LNCaP derived subline; RRID:CVCL_ZC87) were provided by Alan K. Meeker and Jun Luo (Johns Hopkins University, Baltimore, Maryland, USA). Knockout was conducted by transfecting the cells with non‐silencing or TF siRNA (SmartPool, Horizon) using RNAiMax (Thermofisher) according to the manufacturer's protocol. Cells were lysed with RIPA buffer (Pierce) supplemented with protease inhibitor cocktail (Roche). Protein extracts (25 μg) were separated on 7% NuPAGE Tris‐Acetate gel (Invitrogen) by electrophoresis and subsequently transferred onto Immobilon‐P PVDF membranes of 0.45 μm pore size (Millipore).Tumor cell lines (PC‐3, 786‐O, HCT 116, BxPC‐3) were fixed in 10 ml 4% formaldehyde in PBS at 4°C, O/N, followed by incubation in fresh 4% formaldehyde at 65°C for 3 min. 4% agarose in 1× Tris‐acetate‐EDTA (TAE) buffer was added to the cell suspension (1:1) and transferred to a 24‐well plate, incubated at 4°C, O/N and fixed in 4% formaldehyde at 4°C, O/N. Agar cell blocks were embedded in paraffin.

#### 2.1.2 Kidney tissue sample

Freshly excised normal kidney tissue (VUmc) was directly subjected to standard formalin fixation and paraffin embedding. TF expression was assessed at sequential time intervals ranging from 0–10 months on freshly cut slides, using the validated TF IHC protocol (Method 1, with a primary antibody concentration of 2.5 μg/ml HTF‐1).

#### 2.1.3 Tissue microarrays

Tissue microarrays (TMA) were purchased from US Biomax Inc (Derwood, MD, USA) (Suppl. Table S1). These included de‐identified specimens taken from patients with bladder, breast, cervical, colon, prostate (hormone‐resistant), ovarian, endometrial, pancreatic and esophageal cancer, HNSCC, NSCLC, and glioblastoma. Patient‐matched specimens (i.e., paired tumor biopsies from the same patient) of primary tumors and lymph node metastases were available for 31 NSCLC patients (Table S1).

#### 2.1.4 Patient‐matched tumor biopsies taken at different time points

Patient‐matched tumor biopsies (i.e., paired tumor biopsies from the same patient) were collected at various time intervals (time point 1 [T1] and time point 2 [T2]) at the ICR and University Hospitals Leuven. Tumor biopsies were formalin fixed and paraffin embedded according to standard protocols applied at the ICR and University Hospitals Leuven. An overview of the patient‐matched tumor biopsies, per cancer type, and collected at various time intervals is shown in Table S2. Relevant clinical and treatment‐related information was retrieved from the patients' medical records and summarized in Table S3.

### 2.2 Immunohistochemistry (IHC)

#### 2.2.1 Antibody validation

The mouse anti‐human TF antibody, clone HTF‐1 (BD Biosciences Cat# 550252, RRID:AB_393557), was validated using IHC on DU145 cells, with and without siRNA knockdown of TF (Figure S1A), and Western Blot analysis on multiple cell lines known to express (DU145, and to a lesser extent PC‐3) or not express (LNCaP, LNCaP95,) TF, utilizing previously described methods^16^ (Figure S1B). Target specificity of clone HTF‐1 was further confirmed by IHC analysis on formalin‐fixed, paraffin‐embedded (FFPE) cell pellets of tumor cell lines with no (A549), low (HeLa, U‐87 MG), or high (A431) TF mRNA levels, and on samples from normal and tumor tissues (Figure S1D–G).

#### 2.2.2 IHC staining methods

##### IHC staining method 1

Patient‐matched tumor biopsies from bladder (*n* = 2), cervical (*n* = 1), lung (*n* = 2), esophageal (*n* = 3), prostate (*n* = 26), ovarian (*n* = 9) cancer were stained according to IHC staining method 1. All reagents used in this method, except the primary antibody, were purchased from Ventana Medical Systems Inc. (VMSI, Tucson, AZ, USA). Paraffin sections (4 μm thickness) on glass slides were transferred to a Discovery Ultra autostainer (VMSI), deparaffinized and rehydrated. Antigen retrieval was performed by incubating the sections in cell conditioning 1 (CC1) buffer at 95°C for 32–40 min. Endogenous peroxidase was quenched with Ventana inhibitor CM for 8 min. Subsequently, sections were incubated with 3 μg/ml mouse anti‐human TF antibody (clone HTF‐1; BD Biosciences Cat# 550252, RRID:AB_393557) in diluent 95 119 (VMSI) at 37°C for 20 min, followed by OmniMap mouse‐HRP multimer at 37°C for 16 min, amplified with Discovery HQ‐HRP at 37°C twice for 16 min and developed with 3,3′‐diaminobenzidine (DAB) at RT for 5 min. Between incubation steps, sections were thoroughly washed with reaction buffer. Sections were counterstained with hematoxylin II for 16 min to detect individual cells/nuclei and embedded in glycergel.

##### IHC staining method 2

Patient‐matched tumor biopsies from cervical (*n* = 10), ovarian (*n* = 17) and endometrial (*n* = 8) cancer were stained according to IHC staining method 2. Paraffin sections (3 μm thickness) were deparaffinized and rehydrated. Antigen retrieval was performed by heating the slides in citrate buffer (pH 6; TCS Bioscience Ltd, Buckingham, UK) using microwave irradiation (800 W) for 18 min. After cooling to RT, slides were transferred to the Launch IHC Autostainer (model i6000; BioGenex, Fremont, CA, USA). Endogenous peroxidase was quenched in 3% H_2_O_2_ (Sigma‐Aldrich, Zwijndrecht, the Netherlands) at RT for 10 min, and endogenous Fc receptors were blocked for non‐specific antibody binding with Protein block serum‐free (DAKO, Heverlee, Belgium) at RT for 10 min. Subsequently, sections were incubated with 5 μg/ml mouse anti‐human TF (clone HTF‐1; BD Biosciences Cat# 550252, RRID:AB_393557) in REAL antibody diluent (DAKO) at RT for 1 h, followed by undiluted Envision poly‐horseradish peroxidase (HRP) conjugated anti‐mouse/anti‐rabbit IgG polymer at RT for 30 min, and developed with 3,3′‐diaminobenzidine (DAB; both DAKO) at RT for 5 min. Between incubation steps, sections were thoroughly washed with Tris‐buffered saline (pH 7.4) supplemented with 0.2% (v/v) Tween 20 (TBST; Sigma‐Aldrich). Nuclei were counterstained with Harris' hematoxylin (TCS Bioscience Ltd) at RT for 3 min. Finally, the slides were embedded in DPX mounting medium (Merck).

#### 2.2.3 Correlation between IHC method 1 and 2

The methodological differences in the two IHC methods are summarized in Table S4. Reproducibility and assay dynamic range of both methods were verified using formalin‐fixed paraffin‐embedded (FFPE) tumor cell lines with known TF expression. The IHC methods showed high reproducibility and a comparable assay range (Figure S2).

#### 2.2.4 Immunohistochemical staining of TMAs and normal kidney biopsies

All tumor TMAs and normal kidney biopsies were stained according to Ventana's TF IHC protocol, which is similar to IHC method 1, with minor adjustments: incubation with CC1 buffer was performed for 64 minutes instead of 36 minutes, incubation with mouse anti‐human TF antibody was performed with 2.5 μg/ml instead of 3 μg/ml and staining was performed on a Ventana BenchMark slide stainer.

#### 2.2.5 Immunohistochemical staining of tumor biopsies

Freshly cut FFPE tumor tissue sections were stained according to IHC staining method 1 or 2, as indicated above, which were verified for reproducibility and assay range (Figure S2). To exclude potential impact of storage of FFPE tissue blocks on TF protein/epitope stability, it was confirmed via IHC that there was no change in TF staining in an archival FFPE tissue block of healthy kidney tissue for up to at least 10 months (Figure S3).

### 2.3 Image acquisition

Immunostained sections were digitized with an Aperio AT2 slide scanner (Leica Biosystems, Wetzlar, Germany) or an Axio Scan.Z1 (Zeiss, Oberkochen, Germany) slide scanner at 20× magnification.

### 2.4 Manual scoring

Tumor cells in whole tissue scans were identified by histopathological features. Digital images were evaluated manually by one of two board‐certified anatomic pathologists (from Ventana or VUmc), who were blinded for the details of the biopsies. Both TF staining intensity and percentage of tumor cells staining at each intensity were scored on a semi‐quantitative integer scale. Intensity: 0 (negative), 1+ (low), 2+ (medium), or 3+ (high); percentage: from 0% to 100%, at a resolution of 1%–10% scoring intervals. In tumor samples, only viable malignant cells were scored. The membrane and cytoplasm subcellular distribution of TF expression was determined for TMA samples (Ventana), while overall cellular distribution of TF expression was determined in patient‐matched biopsies (either matched primary tumor and metastasis, or patient‐matched samples collected at various time intervals), tumor cell lines, and normal kidney biopsies (VUmc). TF prevalence was defined as the percentage of patients (cases) that showed TF expression (intensity low [1+], medium [2+], or high [3+]) in ≥10% of the tumor cells in their tumor biopsies. TF expression was scored by modified histologic score (H‐score) method with score ranging from 0 (completely negative) to 300 (100% of tumor cells strongly positive). The H‐score was calculated according to Equation (1):

(1) $$\mathrm{TF}H-\text{score}=(0\times \left[\%\text{cells with intensity of}\;0\right]+1\times \left[\%\text{cells with intensity}\;1+\right]+2\times \left[\%\text{cells with intensity}\;2+\right]+3\times \left[\%\text{cells with intensity}\;3+\right]).$$

### 2.5 Statistical analysis

The Wilcoxon matched‐pair signed rank test was used to determine statistically significant differences in TF H‐scores of matched tumor biopsies across patient populations (resected from different locations or at different time points), using GraphPad Prism (http://www.graphpad.com, RRID:SCR_002798, version 7.2). Grouped analyses were performed on results from all cancer types combined, and separate analyses were performed per cancer type.

To assess whether the relation between TF expression levels in sequential biopsies taken from the same patient was impacted by the time interval between resection of the tumor biopsies (Time 1 vs. Time 2, or T1 vs T2), the variation in TF H‐scores between T1 and T2 was quantified by subtracting the log(TF H‐score at T1) from the log(TF H‐score at T2).

### 2.6 TF mRNA expression in solid tumors

The Cancer Genome Atlas (TGCGA, http://cancergenome.nih.gov, RRID:SCR_003193) was consulted for TF mRNA expression in solid tumors. From this database, analyzed mRNA samples of glioblastoma (GBM; *n* = 186), TNBC (*n* = 116), prostate cancer (PRAD, *n* = 502), non‐TNBC breast cancer (*n* = 612), NSCLC (LUAD and LUSC, *n* = 1041), pancreatic cancer (PAAD; *n* = 179), HNSCC (HNSC, *n* = 522), cervical cancer (CESC; *n* = 306), endometrial cancer (UCEC, *n* = 551), esophageal cancer (ESCA, *n* = 185), bladder cancer (BLCA, *n* = 411), ovarian cancer (OV, *n* = 430), and colon cancer (COAD; *n* = 472) patients were used. TF mRNA expression (FPKM) in solid tumors was retrieved from the TCGA database using OncoLand (version 10.0.1.72, QIAGEN) and analyzed using GraphPad Prism (version 7.2).

## 3 RESULTS

### 3.1 Prevalence and cellular localization of TF expression in solid tumors

To systematically assess and compare the prevalence of TF expression in solid tumors, IHC analysis using validated reagents and methods was performed on tumor tissue and TMAs that included specimens of a broad range of solid cancer types including gynecological, urological, breast, gastrointestinal, head and neck, non‐small cell lung, and glioblastoma.

**FIGURE 1:**
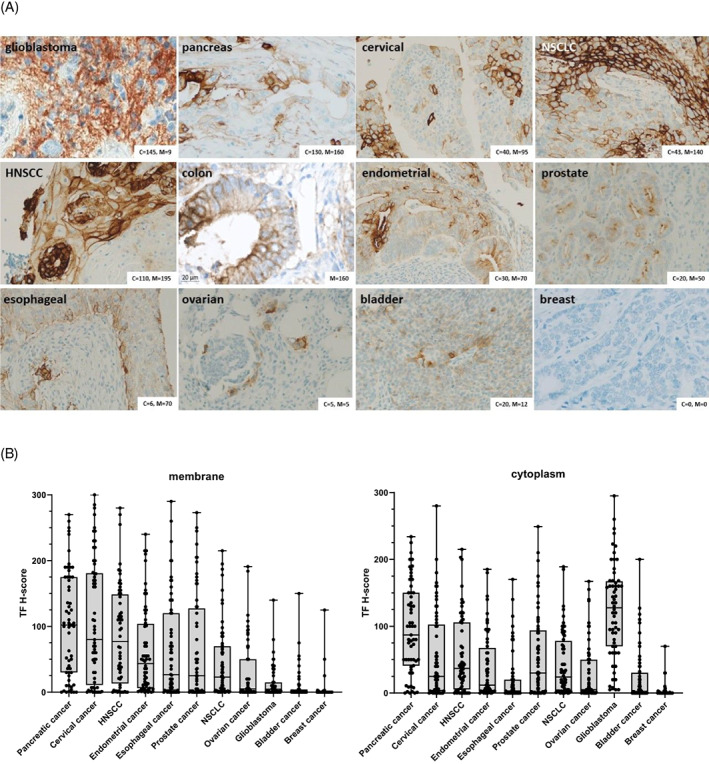
TF immunostaining in solid tumor TMAs. Solid tumor TMAs (Table S1) were stained with mouse anti‐human TF (clone HTF‐1; 3 μg/ml; brown). Nuclei were counterstained with hematoxylin (blue). (A) Pictures show representative examples of TF staining in solid tumor biopsies with different intensities and subcellular localizations. Original magnification: 20×. TF staining intensity (scored on a semi‐quantitative integer scale from 0 [negative] to 3+ [high]) and the percentage of tumor cells staining positively at each intensity level was scored by a certified anatomical pathologist. H‐scores for cytoplasmic and membranous TF were calculated according to Equation (1), and scores are indicated in each panel ((B) indicates the TF H‐score for cytoplasmic expression, M indicates TF H‐score for membrane expression). TF staining in: (upper panel, left to right) glioblastoma tumor biopsy (GL8063, core D3), pancreatic tumor biopsy (PA961b, core D2), cervical tumor biopsy (CXC1021, core B4), NSCLC tumor biopsy (LC1021, core F8), (middle panel, left to right) HNSCC tumor biopsy (HN803b, core G4), colon tumor biopsy (CO2081, core A6; cytoplasmic TF H‐scores were not determined), endometrial tumor biopsy (EMC1021, core C9), prostate tumor biopsy (PR803b, core A8), (lower panel, left to right) esophageal tumor biopsy (ES1021, core A8), ovarian tumor biopsy (OV803b, core B7), bladder tumor biopsy (BL802, core A4), and breast tumor biopsy (BRC1021, core C7). (B) Distribution of cytoplasmic and membrane TF H‐scores in solid tumor TMAs. Left: Distribution of membrane TF H‐scores. Right: Distribution of cytoplasmic TF H‐scores. Data shown for each cancer type are individual TF H‐scores (dots), with boxes extending from the 25th to 75th quartiles, in which the median TF H‐score is indicated and whiskers showing minimum and maximum TF H‐scores

TF‐positive tumors (defined as tumors expressing TF in >10% of the tumor cells) were identified in all malignancies studied, with the majority of malignancies showing expression in more than half of the biopsies assessed. There was heterogeneity in percentage of cells expressing TF and staining intensity (via H‐score) between, but also within the different tumor types (Table 1, Figure 1). The highest prevalence of TF expression (≥75% TF‐positive tumor biopsies, with either membranous or cytoplasmic TF expression) was found in glioblastoma, pancreatic cancer, cervical cancer, colon cancer, NSCLC, and HNSCC. The frequency of TF‐positive biopsies was lowest in breast cancer (10%), with some enrichment in the triple‐negative breast cancer (TNBC) population (35%) (Table 1).

**FIGURE 2:**
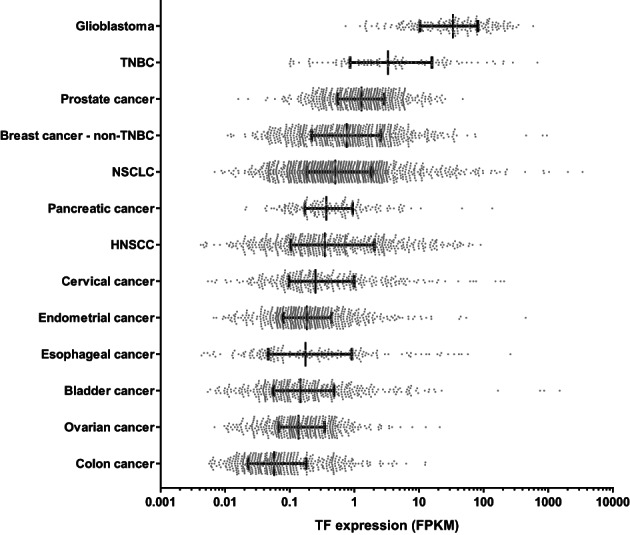
TF mRNA expression in solid tumors. The median TF mRNA expression ranged from 0.05747 to 33.49 fragments per kilobase of exon per million mapped reads (FPKM), with high TF expression (FPKM >0.2) in glioblastoma, breast cancer (both TNBC and non‐TNBC), prostate cancer, NSCLC, pancreatic cancer, HNSCC, and cervical cancer. Low TF gene expression (FPKM <0.2) was observed in endometrial cancer, esophageal cancer, bladder cancer, ovarian cancer, and colon cancer. The samples included glioblastoma (GBM; *n* = 186), triple‐negative breast cancer (TNBC, selected from BRCA, *n* = 116), prostate cancer (PRAD, *n* = 502), non‐TNBC breast cancer (selected from BRCA, *n* = 612), NSCLC (LUAD and LUSC, *n* = 1041), pancreatic cancer (PAAD; *n* = 179), HNSCC (HNSC, *n* = 522), cervical cancer (CESC; *n* = 306), endometrial cancer (UCEC, *n* = 551), esophageal cancer (ESCA, *n* = 185), bladder cancer (BLCA, *n* = 411), ovarian cancer (OV, *n* = 430), and colon cancer (COAD; *n* = 472). Data are represented as median ± interquartile range. Statistical analysis was performed using a Kruskal‐Wallis test and showed significant differences in TF mRNA expression between cancer types (*p* < .0001)

In the majority of cancers, TF expression was observed on the plasma membrane, in addition to cytoplasmic staining in some cancer types (Figure 1). TF staining was predominantly cytoplasmic in bladder cancer and glioblastoma, with no expression in the nuclei. Although only membrane‐expressed TF can form TF:FVIIa complexes, it has been shown that TF recycles quickly between the membrane and the cytoplasm.^17^ Analysis of TF mRNA expression using The Cancer Genome Atlas (TCGA) database confirmed TF expression across solid cancer types, with heterogeneity between and within cancer types (Figure 2).

Together, these results show that TF is abundantly expressed across solid tumors, and expression is observed both on the cell membrane and in the cytoplasm of tumor cells.

### 3.2 Temporal dynamics of TF in solid tumor biopsies

To investigate the temporal dynamics of TF expression, IHC was performed on matched tumor biopsies from patients with cervical (*n* = 11), ovarian (*n* = 26), prostate (*n* = 26), endometrial (*n* = 8), lung (*n* = 2), bladder (*n* = 2), and gastro‐esophageal cancer (*n* = 3), collected at two different timepoints (T1 and T2) with intervals ranging from 4–150 months (Tables S2 and S3).

**FIGURE 3:**
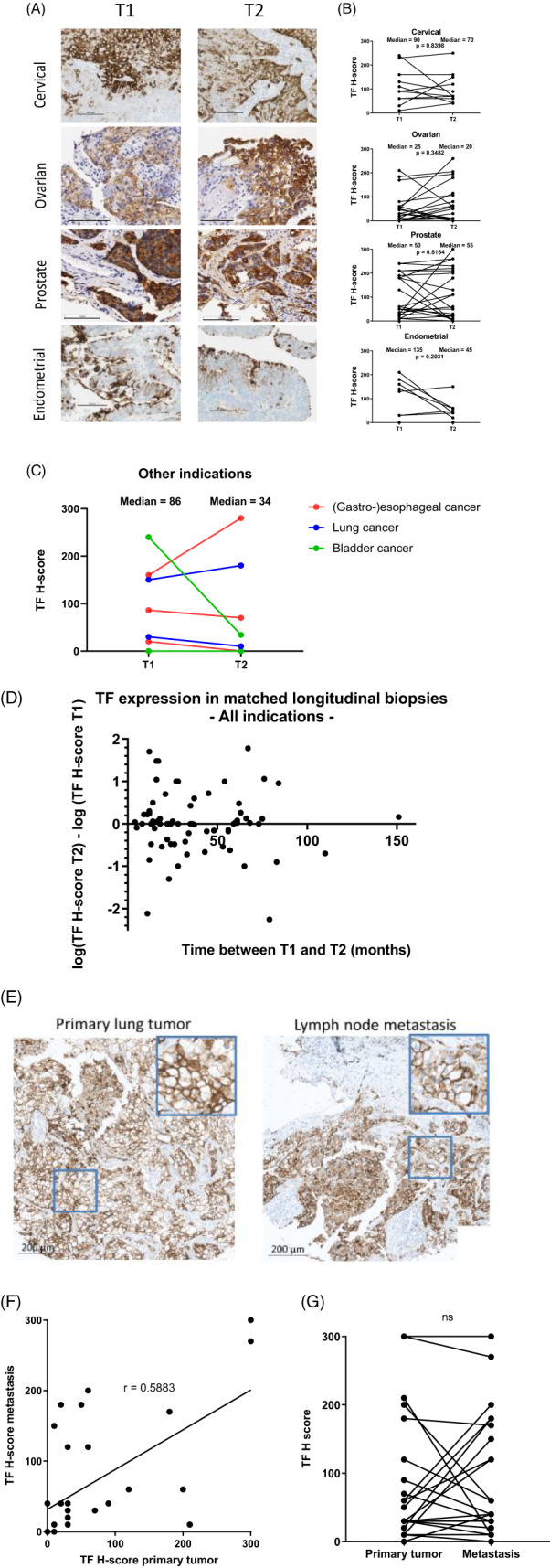
TF immunostaining in patient‐matched tumor biopsies collected at various time intervals and of primary tumors and lymph node metastases from NSCLC patients. TF staining (mouse anti‐human TF [clone HTF‐1; 3 μg/ml; brown]; nuclei were counterstained with hematoxylin [blue]) was performed on patient‐matched T1 and T2 tumor biopsies of cervical (*n* = 11), ovarian (*n* = 26), prostate (*n* = 26), endometrial (*n* = 8), esophageal (*n* = 3), lung (*n* = 2) and bladder (*n* = 2) cancer patients (A–D)), or on matched biopsies from primary lung tumors and lymph node metastasis from NSCLC patients (*n* = 31; TMA‐LC814a) (E–G). Pictures show representative examples of TF staining in primary tumor biopsies (A) and a primary tumor biopsy with a patient‐matched lymph node metastasis (E). Original magnification: 20×. Scale bar represents 100 mm (A) or 200 mm (E). TF staining intensity (scored on a semi‐quantitative integer scale from 0 (negative) to 3+) and the percentage of tumor cells staining positively at each intensity level was scored by a certified anatomical pathologist. H‐scores for TF were calculated according to Equation (1). (A) TF staining (brown) was observed in archival T1 (left panels) and T2 (right panels) patient‐matched tumor biopsies. TF expression was heterogeneous and tumor cells with and without TF expression were observed. Representative examples are shown of a cervical cancer patient (4‐month time interval; TF H‐scores 230 (T1) and 250 (T2)); an ovarian cancer patient (24‐month time interval; TF H‐scores 183 (T1) and 205 (T2)); a prostate cancer patient (60‐month time interval; TF H‐scores 240 (T1) and 260 (T2)); and an endometrial cancer patient (25‐month time interval; TF H‐scores 130 (T1) and 150 (T2)). (B) Analysis of differences in TF H‐scores in archival T1 and T2 patient‐matched tumor biopsies from patients with cervical, ovarian, prostate, endometrial cancer. Statistical analysis was performed using a Wilcoxon signed rank test and indicated no significant differences in TF H‐scores between T1 and T2 in all analyzed groups. (C) Analysis of differences in TF H‐scores in matched T1 and T2 tumor biopsies in three cancer types ([gastro]‐esophageal, lung and bladder) grouped together. (D) Correlation between time interval of the tumor biopsies and the variation in TF H‐scores in patient‐matched T1 and T2 tumor biopsies. The variation in TF H‐scores for each patient was calculated by subtracting the log(TF H‐score at T1) from the log(TF H‐score at T2), and was plotted against the time interval between the dissection of the T1 and T2 tumor biopsies. (E) Representative example of TF staining in biopsies of primary lung tumor (LC814a, core B10, TF H‐score: 300) and lymph node metastasis (LC814a, core F10, TF H‐score: 270) of a NSCLC patient. (F) Linear regression of TF H‐scores of matched primary tumor biopsies and lymph node metastases. Statistical analysis was performed using a non‐parametric Spearman correlation showed a significant correlation in the TF H‐scores of primary tumor biopsies with lymph node metastasis (*p* = .0005; *r* = .5883). (G) Analysis of differences in TF H‐scores in matched primary lung tumors and lymph node metastases. Statistical analysis was performed using a Wilcoxon signed rank test and indicated no significant differences in TF H‐scores of matched primary tumor biopsies and lymph node metastasis

Tumor TF expression (H‐score) was generally comparable between the first and second biopsy in patients with cervical, ovarian, and prostate cancer (Figure 3A,B), although differences in H‐score were observed between T1 and T2 for individual patients. In biopsies from patients with endometrial cancer, H‐scores appeared to be lower in T2 than in T1 samples. However, sample size (*n* = 8) was not sufficiently large to draw firm conclusions to whether the differences in TF expression indeed reflect changes due to time or treatment, or reflect tumor heterogeneity. No trend for variation in TF expression over time was observed in patient‐matched tumor biopsies of the remaining cancer types that were grouped together because they had insufficient numbers for statistical analysis (i.e., gastro‐esophageal, lung, and bladder cancer) (Figure 3C).

Variation in TF H‐scores between T1 and T2 was not dependent on the time interval between the biopsies (Figure 3D) and appeared to be unrelated to disease progression in prostate, cervical, ovarian, and endometrial cancer patients as depicted by early (E; stage 1 and 2) and advanced (A; stage 3 and 4) stages of disease (Figure S4). Similarly, treatment received between T1 and T2, including chemotherapy, radiotherapy, or hormone treatment (Table S3), did not have a significant effect on TF expression at the population level (data not shown). Although TF expression was not significantly different between T1 and T2 samples per cancer type and appeared to be independent of disease progression or treatment, differences were observed for some patients.

### 3.3 Spatial dynamics of TF in tumor biopsies and matched lymph node metastases

TF expression was assessed in primary NSCLC tumors and patient‐matched lymph node metastases that were resected on the same day (*n* = 31; example in Figure 3E). TF expression in primary tumors showed a clear correlation with TF expression in tumor metastases across the studied group (*p* = .0005; *r* = .5883; Figure 3F), although there were some differences in TF expression between primary and metastatic lesions for individual patients. Correspondingly, there was no significant difference in TF H‐score between primary tumor samples (median TF H‐score = 30) and matched lymph node metastases (median TF H‐score = 30) (Figure 3G). These results indicate that in general TF expression in NSCLC is stable upon metastatic dissemination. Taken together, these results indicate that tumor cell TF expression in solid tumors is generally stable over time, and independent of disease progression or treatment regimen, and independent of tumor dissemination as shown by concordance in expression between patient‐matched primary and metastatic lesions.

## 4 DISCUSSION

TF is aberrantly expressed in a broad range of solid tumors compared to normal tissue,^8^ and has been associated with poor prognosis.^12^, ^13^, ^18^ Here we determined the prevalence, cellular localization, and the spatial and temporal expression patterns of TF in a broad panel of solid tumor biopsies taken at various time points and stages of disease, and from primary and metastatic lesions, using validated reagents and IHC methods.

The results in this manuscript indicate that TF expression in tumor cells is generally stable over time, is independent of disease progression or treatment regimen, and independent of tumor dissemination. These results may appear counter‐intuitive as the literature suggests that TF expression may be increased with more advanced stages of disease, as a prothrombotic state typically characterizes more advanced malignancies^19^ and is a significant cause of patient death.^20^ However, the reported correlation between TF expression and advanced disease is almost exclusively based on TF expression analysis in primary tumors. Here we sought to capture the changes in TF expression from a single patient in early and upon progression in more advanced stages of disease. The data show that while some patients do have changes in their TF expression over time, there is no statistical change at a population level in the samples tested. It is possible that TF expression in the primary lesion is related to a more aggressive cancer type or may reflect the complexity of TF biology with diverse functions in cancer beyond coagulation. On the other hand, stromal TF expression, which was not taken into account in the present study, may also contribute to an enhanced prothrombotic state in advanced malignancies.

A technical limitation of our study, and of biopsies in general, is that patient‐matched biopsies collected at various time intervals were often not the same size or were obtained from a different anatomical location. The collection of multiple or equally‐sized biopsies for research purposes without a direct benefit for the patient is limited by safety risks associated with the procedure,^21^, ^22^ and in some patients the primary tumor site was completely removed during the initial resection.

Given the mentioned restriction, this study is the first detailed and systematic analysis of the prevalence, cellular localization, and spatial and temporal expression of TF in solid tumors. Normal tissues were not scored, because TF typically has low expression in most tissues as described previously.^5^, ^6^ We show that TF is expressed in tumor cells across a broad range of solid cancer types, with differences in TF expression prevalence and staining intensity between various solid tumor specimens. Distinctive patterns were observed across solid tumors, with pancreatic cancer, cervical cancer and HNSCC standing out with, predominantly membranous, TF expression in at least 75% of the cases. Interestingly we observed low prevalence of TF expression in breast, ovarian, and bladder cancer which are in discordance with previous reports.^23^, ^24^, ^25^ This discordance may be a result of differences in methodology (e.g., detection antibodies or assay parameters), the selected patient population, the composition of the scored tissue samples, biological differences, or even publication bias, since negative results are less likely to be published. The present study restricted analysis of TF expression to tumor cells and did not take into account expression on other cells in the tumor microenvironment (e.g., stromal cells), which has been previously described in some solid tumors.^26^

TF H‐scores in primary tumors showed a significant correlation with patient‐matched metastasis. At the population level, TF H‐scores were generally stable over time irrespective of disease progression or treatment, although differences in H‐scores between biopsies taken at different timepoints were observed for individual patients.

The broad expression across solid cancers makes TF a relevant therapeutic target and supports further investigation of TF‐targeting agents across multiple tumor subtypes and various stages of disease. These data suggest that TF might be considered as a biomarker for various solid tumors, but future large‐scale studies are warranted.

## AUTHOR CONTRIBUTIONS

**Johann S. de Bono:** Conceptualization (equal); data curation (equal); supervision (equal); writing – review and editing (equal). **Jeffrey R. Harris:** Data curation (equal); formal analysis (equal); methodology (equal); project administration (equal); writing – original draft (equal); writing – review and editing (equal). **Saskia M. Burm:** Formal analysis (equal); project administration (equal); writing – original draft (equal); writing – review and editing (equal). **Adriaan Vanderstichele:** Data curation (equal); investigation (equal); methodology (equal); writing – review and editing (equal). **Mischa A. Houtkamp:** Conceptualization (equal); data curation (equal); formal analysis (equal); investigation (equal); methodology (equal); writing – review and editing (equal). **Saida Aarass:** Investigation (equal); methodology (equal); validation (equal). **Ruth Riisnaes:** Data curation (equal); formal analysis (equal); investigation (equal); validation (equal); writing – review and editing (equal). **Ines Figueiredo:** Data curation (equal); formal analysis (equal); investigation (equal); writing – review and editing (equal). **Daniel Nava Rodrigues:** Investigation (equal); validation (equal); writing – review and editing (equal). **Rossitza Christova:** Formal analysis (equal); investigation (equal); validation (equal); writing – review and editing (equal). **Siel Olbrecht:** Investigation (equal); writing – review and editing (equal). **Hans W.M. Niessen:** Data curation (equal); formal analysis (equal); investigation (equal); methodology (equal); validation (equal); writing – review and editing (equal). **Sigrid R. Ruuls:** Data curation (equal); formal analysis (equal); writing – original draft (equal); writing – review and editing (equal). **Danita H. Schuurhuis:** Data curation (equal); formal analysis (equal); writing – original draft (equal); writing – review and editing (equal). **Jeroen J. Lammerts van Bueren:** Conceptualization (equal); data curation (equal); formal analysis (equal); writing – original draft (equal); writing – review and editing (equal). **Esther C.W. Breij:** Conceptualization (equal); formal analysis (equal); supervision (equal); writing – original draft (equal); writing – review and editing (equal). **Ignace Vergote:** Conceptualization (equal); supervision (equal); writing – review and editing (equal).

## CONFLICT OF INTEREST

J.S.B., A.V., R.R., I.F., D.N.R., R.C., S.O., H.N., and I.V. performed this research under a sponsored research agreement with Genmab. J.R.H, S.M.B., M.A.H., S.A., S.R.R., D.H.S., J.J.L.B., and E.C.W.B. are/were employees of Genmab and own Genmab warrants and/or stock.

## ETHICS STATEMENT

At The Institute of Cancer Research (ICR), London and University Hospitals Leuven, biopsies were taken according to biobanking protocols approved by the respective local ethical committees. The Ethics Committee Research UZ/KU Leuven (EC Research) approved of the project in the meeting of 20‐May‐2016 (reference letter S58847). Patients referred for phase 1 trial participation at the Royal Marsden Hospital site were consented to a tumor molecular characterization protocol approved by the Royal Marsden Clinical Research and Ethics committees (CCR3171).

## PATIENT CONSENT STATEMENT

All patients had provided informed consent for use of their tumor tissue for scientific research.

## Supporting information


**Appendix S1**. Supporting Information.

## Data Availability

The data that support the findings of this study are available from the corresponding author upon reasonable request.
