# Correction to “Systematic Study of Tissue Factor Expression in Solid tumors”

**DOI:** 10.1002/cnr2.2143

**Published:** 2024-07-23

de Bono JS, Harris JR, Burm SM, Vanderstichele A, Houtkamp MA, Aarass S, Riisnaes R, Figueiredo I, Nava Rodrigues D, Christova R, Olbrecht S, Niessen HWM, Ruuls SR, Schuurhuis DH, Lammerts van Bueren JJ, Breij ECW, Vergote I. Systematic study of tissue factor expression in solid tumors. *Cancer Reports*. 2023;6:e1699.

In Table 1 for the data reported in literature for HNSCC, the TF prevalence (%) should read “58%–100%.” The two references mentioned (Refs. 18 and 19 in the Supporting Information file; Supporting Information References) should read:

18. Christensen A, Kiss K, Lelkaitis G, Juhl K, Persson M, Charabi BW, Mortensen J, Forman JL, Sorensen AL, Jensen DH, Kjaer A, von Buchwald C. Urokinase‐type plasminogen activator receptor (uPAR), tissue factor (TF) and epidermal growth factor receptor (EGFR): tumor expression patterns and prognostic value in oral cancer. *BMC Cancer*. 2017;17: 572.

19. Wojtukiewicz MZ, Zacharski LR, Rucinska M, Zimnoch L, Jaromin J, Rozanska‐Kudelska M, Kisiel W, Kudryk BJ. Expression of tissue factor and tissue factor pathway inhibitor in situ in laryngeal carcinoma. *Thromb Haemost*. 1999;82: 1659–1662.

We apologize for this error.

