# Supplementary material for: Systematic study of tissue factor expression in solid tumors
Source: Cancer Rep (Hoboken). 2022 Aug 13;6(2):e1699. doi: 10.1002/cnr2.1699 (PMC9940005; doi:10.1002/cnr2.1699)
Supplement: Supplementary file 1 — Appendix S1. Supporting Information. [file CNR2-6-e1699-s001.docx]

Supplementary Information

Systematic study of tissue factor expression in solid tumors

Supplementary Tables

Table S1. Overview of the solid tumor TMAs

| **Cancer type** | **N**  **(patients)** | **N (cores)** | **Tumor type** | **TMA code**  **(Biomax)^1^** |
| --- | --- | --- | --- | --- |
| Bladder cancer | 58 | 58 | TCC | BL802 |
| Breast cancer | 40 | 40 | IDC, ILC | BRC1021 |
| Breast cancer (triple negative; TNBC) | 46 | 46 | IDC, ILC, lobular ductal mixed carcinoma, medullary carcinoma | BR487a |
| Cervical cancer | 61 | 61 | AC, SQCC, endometroid AC | CXC1021 |
| Colon cancer | 34 | 34 | AC | CO2081 |
| HNSCC | 60 | 60 | SQCC | HN803b |
| Prostate cancer (HR) | 60 | 60 | AC | PR803b |
| Ovarian cancer | 60 | 60 | AC | OV803b |
| Endometrial cancer | 60 | 60 | AC, ASQC | EMC1021 |
| NSCLC | 60 | 60 | AC, SQCC, ASQC, BA, NE, other^2^ | LC1021 |
| NSCLC  (Matched primary tumor and metastasis) | 31 | 62 | AC, SQCC | LC814a |
| Pancreatic cancer | 59 | 59 | AC, NE, carcinoid, other^3^ | PA961b |
| Esophageal cancer | 62 | 62 | SQCC | ES1021 |
| Glioblastoma | 60 | 60 | GBM, pleomorphic GBM | GL803c, GL806f |

AC: adenocarcinoma; ASQC: adenosquamous carcinoma; BA: bronchioloalveolar carcinoma; GBM: glioblastoma; HNSCC: head and neck squamous cell carcinoma; HR: hormone refractory; IDC: infiltrating ductal carcinoma, ILC: infiltrating lobular carcinoma; NE: neuroendocrine tumor; NSCLC: non-small cell lung cancer; SCLC: small cell lung cancer; SQCC: squamous cell carcinoma; TCC: transitional cell carcinoma; TMA: tissue microarray; TNBC: triple-negative breast cancer

^1^Detailed information on tumor cores is accessible on the website of the provider (US Biomax Inc)

^2^Other lung TMA samples (n=1): papillary carcinoma, clear cell carcinoma, and carcinoid

^3^Other pancreatic TMA samples (n=1): undifferentiated carcinoma and mixed acinar-neuroendocrine carcinoma

Table S2. Overview of patient-matched tumor biopsy pairs collected at various time intervals and median TF H-scores at sampling interval time (T1 - T2)

| **Cancer type** | **Sample size** | **TF H-score T1 (Median)** | **TF H-score T2 (Median)** | **P value^1^** | **TF H-score T1**  **(Mean ± SEM)** | **TF H-score T2**  **(Mean ± SEM)** | **Time interval in months (Mean ± SEM)** |
| --- | --- | --- | --- | --- | --- | --- | --- |
| Cervix | 11 | 90 | 70 | 0.8398 | 110 ± 23 | 102 ± 19 | 21.6 ± 3.8 |
| Ovary | 26 | 25 | 20 | 0.3482 | 42 ± 11 | 57 ± 15 | 34 ± 4 |
| Prostate | 26 | 50 | 55 | 0.8164 | 94 ± 17 | 107 ± 21 | 50.5 ± 6.4 |
| Endometrium | 8 | 135 | 45 | 0.2031 | 110 ± 28 | 53 ± 16 | 33.5 ± 10.4 |
| Esophagus (gastro) | 3 | 86 | 70 | N/A | 89 ± 40 | 117 ± 84 | 13.3 ± 5.2 |
| Lung (NSCLC) | 2 | 90 | 95 | N/A | 90 ± 60 | 95 ± 85 | 37 ± 24 |
| Bladder | 2 | 120 | 17 | N/A | 120 ± 120 | 17 ± 17 | 17.5 ± 5.5 |

NSCLC: non-small cell lung cancer; SEM: standard error of the mean

^1^Statistical analysis: Wilcoxon matched pairs signed rank test

Table S3. Patient characteristics, including treatment during study, and TF H-scores of patient-matched tumor biopsies collected at various time intervals

Relevant clinical information as retrieved from the patients’ medical records. Disease stage was determined according to the 7^th^ edition of the Union for International Cancer Control (UICC)/American Joint Committee on Cancer (AJCC) staging system and the International Federation of Gynecology and Obstetrics (FIGO) staging system (gynecological cancers).

During the interval between resection of the patient-matched biopsies (between T1 and T2), most patients received a form of standard therapy that included chemotherapy, radiotherapy, or hormone treatment. Most patients were treated with a cisplatin- and taxane-based chemotherapy regimen. All cervical and ovarian cancer patients received chemotherapy either as single therapy (n = 2 for cervical, n = 25 for ovarian cancer) or in combination with radiotherapy (n = 9 for cervical, n = 1 for ovarian cancer). All prostate cancer patients received hormone treatment in combination with chemotherapy (n = 18), radiation (n = 3), or both (n = 5). Of endometrial cancer patients (n = 6), four patients did not receive any treatment after tumor resection, one patient received chemotherapy and another patient received radiotherapy.

| Gender  (M/F) | Cancer type | UICC /AJCC or FIGO stage at diagnosis^1^ | Treatment until date of follow up biopsy^2^ | Time interval  (T1 →T2)  (months) | Disease course  (T1 → T2)^3^ | TF H-score^4^  T1 | TF H-score^4^  T2 |
| --- | --- | --- | --- | --- | --- | --- | --- |
| F | Cervix | IIIA | C (P) + RT | 21 | AA | 150 | 30 |
| F | Cervix | IVB | C (P+T) | 37 | AA | 10 | 40 |
| F | Cervix | IB1 | C (P+T) | 4 | EE | 250 | 230 |
| F | Cervix | IIB | C (P+T) + RT* | 15 | EA | 70 | 90 |
| F | Cervix | IIIB | C (P) + RT | 17 | AA | 70 | 60 |
| F | Cervix | IVA | C (P) + RT | 14 | AA | 60 | 60 |
| F | Cervix | IIA | C (P+T) + RT | 18 | EA | 120 | 90 |
| F | Cervix | IIB | C (P) + RT | 13 | EA | 160 | 160 |
| F | Cervix | IIB | C (P) + RT | 48 | EA | 90 | 130 |
| F | Cervix | IB2 | C (P+T) + RT | 19 | EA | 70 | 240 |
| F | Cervix | IV | C (P+T) + RT* | 32 | AA | 42 | 110 |
| F | Ovary | IIIC | C (P+T) | 21 | AA | 0 | 50 |
| F | Ovary | IIIC | C (P+T) | 25 | AA | 0 | 60 |
| F | Ovary | IIIC | C (P+T) | 28 | AA | 10 | 0 |
| F | Ovary | IIIC | C (P+T) | 22 | AA | 0 | 0 |
| F | Ovary | IIIC | C (P+T) | 26 | AA | 0 | 30 |
| F | Ovary | IIIC | C (P+T) | 24 | AA | 0 | 0 |
| F | Ovary | IIIC | C (P+T) | 16 | AA | 30 | 0 |
| F | Ovary | IIIC | C (P+T) | 17 | AA | 0 | 60 |
| F | Ovary | IIIC | C (P+T) | 26 | AA | 10 | 30 |
| F | Ovary | IIIC | C (P+T) | 17 | AA | 30 | 0 |
| F | Ovary | IIIC | C (P+T) | 67 | AA | 60 | 0 |
| F | Ovary | IA | C (P) | 62 | EE | 180 | 60 |
| F | Ovary | IA | C (P) | 45 | EA | 260 | 50 |
| F | Ovary | IIIC | C (P+T) | 53 | AA | 60 | 210 |
| F | Ovary | IIB | C (P+T) | 36 | EA | 0 | 0 |
| F | Ovary | IIIC | C (P+T) | 12 | AA | 50 | 0 |
| F | Ovary | IIIC | C (P+T) | 18 | AA | 0 | 0 |
| F | Ovary | I | C (P+T)* | 14 | EA | 205 | 184 |
| F | Ovary | III | C (P+T)* | 43 | AA | 10 | 46 |
| F | Ovary | II | C (P+T) | 14 | EA | 63 | 20 |
| F | Ovary | I | C (P+Tamoxifen) | 14 | AA | 195 | 170 |
| F | Ovary | III | C (P+T)* | 35 | AA | 0 | 0 |
| F | Ovary | IIA | C (P+T)* | 76 | EA | 115 | 10 |
| F | Ovary | IIIC | C (P+T)* | 35 | AA | 80 | 30 |
| F | Ovary | IIC | C (P+T)* | 73 | EA | 10 | 10 |
| F | Ovary | IIC | C (P+T) + RT* | 66 | EA | 107 | 80 |
| M | Prostate | T1bN0M0 | TURP + H (AD) + RT | 11 | EA | 60 | 36 |
| M | Prostate | IV (T4N1M1) | H (AD) + RT + C (T) | 65 | AA | 0 | 10 |
| M | Prostate | TxN1M1 | H (AD) + RT + C (P + T)* | 60 | AA | 260 | 240 |
| M | Prostate | T4N1M0 | H (AD) + C (T)* | 37 | EA | 230 | 240 |
| M | Prostate | T2cNxM1 | H (AD) + C (T) | 27 | AA | 300 | 30 |
| M | Prostate | T3aNxM1 | H (AD) + C (T)* | 12 | AA | 20 | 10 |
| M | Prostate | TxNxM0 | H (AD) + RT | 151 | EA | 260 | 180 |
| M | Prostate | T3cNxM1 | H (AD) + C (T)* | 24 | AA | 20 | 60 |
| M | Prostate | T2aN0M0 | H (AD) + RT* | 34 | EA | 30 | 50 |
| M | Prostate | TxN1M0 | H (AD) + RT + C (T) | 110 | EA | 10 | 50 |
| M | Prostate | T4N1M1b | H (AD) + C (T) | 11 | AA | 0 | 130 |
| M | Prostate | T4N1M0 | H (AD) + C (T)* | 44 | EA | 20 | 30 |
| M | Prostate | T3bN1M1b | H (AD) + C (T) | 28 | AA | 0 | 10 |
| M | Prostate | T3NxM1 | H (AD) + C (P+T)* | 59 | AA | 210 | 210 |
| M | Prostate | T3N2M0 | H (AD) + C (T) | 84 | EA | 180 | 20 |
| M | Prostate | TxNxM1 | H (AD) + C (T)* | 57 | AA | 50 | 210 |
| M | Prostate | T4N1M1 | H (AD) + C (T)* | 18 | AA | 0 | 0 |
| M | Prostate | T4N0M0 | H (AD) + RT + C (T) | 79 | EA | 0 | 180 |
| M | Prostate | T3N1M1 | H (AD) + C (T) | 56 | AA | 20 | 30 |
| M | Prostate | T2NxM1 | H (AD) + C (P+T)* | 56 | AA | 130 | 180 |
| M | Prostate | T1cNxM1b | H (AD) + C (T)* | 63 | AA | 110 | 60 |
| M | Prostate | TxNxM0 | H (AD) + RT + C (P+T) | 68 | EA | 220 | 210 |
| M | Prostate | TxNxM1 | H (AD) + C (T) | 54 | AA | 300 | 30 |
| M | Prostate | T4N1M0 | H (AD) + C (T)* | 61 | EA | 200 | 190 |
| M | Prostate | T4NxM1 | H (AD) + C (T)* | 28 | AA | 50 | 50 |
| M | Prostate | TxNxM1 | H (AD) + C (T)* | 16 | AA | 110 | 10 |
| F | Endometrium | IB | - | 33 | EA | 40 | 210 |
| F | Endometrium | IIIB | RT | 75 | AA | 40 | 30 |
| F | Endometrium | IVA | C (P+T) | 25 | AA | 150 | 130 |
| F | Endometrium | II | - | 22 | EA | 60 | 140 |
| F | Endometrium | IB | - | 8 | EA | 0 | 0 |
| F | Endometrium | IA | - | 13 | EE | 60 | 180 |
| F | Endometrium | IA | - | 9 | EA | 50 | 30 |
| F | Endometrium | IC | - | 83 | EE | 20 | 160 |
|  | Gastro-esophagus | IIIC | C (P) + RT* | 5 | AA | 70 | 86 |
|  | Gastro-esophagus | IV | C (P)* | 23 | AA | 0 | 20 |
|  | esophagus | IV | C (P) + RT* | 12 | AA | 280 | 160 |
|  | Lung | IV | C (P+T) + RT* | 13 | AA | 10 | 30 |
|  | Lung | IV | C (P+T)* | 61 | AA | 180 | 150 |
|  | Bladder | IV | C (P)* | 23 | AA | 0 | 0 |
|  | Bladder | II | C (P)* | 12 | EA | 34 | 240 |

^1^ Staging system according to the Union for International Cancer Control (UICC) and the American Joint Committee on Cancer (AJCC) and FIGO

^2^ C = chemotherapy; P = platins; T = taxanes; RT = radiotherapy; H = hormone therapy; AD = androgen-deprivation therapy; TURP = transurethral resection of the prostate; * = other therapies received in addition to those mentioned

^3^ E = early stage (stage 1 or 2); A = advanced stage (stage 3 or 4)

^4^ TF H-score calculated according to **Equation 1** (main manuscript)

Table S4. Differences between IHC methods

| Protocol feature | Method 1 | Method 2 |
| --- | --- | --- |
| Section thickness | 4 μm | 3 μm |
| Antigen retrieval | CC1 buffer; 95°C; 32-40 min | Citrate buffer (pH 6.0); microwave 800 W; 18 min |
| Quench | Inhibitor CM; 8 min | 3% H_2_O_2_; RT; 10 min |
| Blocking step | - | Protein block serum-free; RT; 10 min |
| Primary antibody | 3 μg/mL mouse a-human TF (clone HTF-1); 37°C; 20 min | 5 μg/mL mouse a-human TF (clone HTF-1); RT; 1h |
| Secondary antibody | OmniMap mouse-HRP; 37°C; 16 min | Envision poly-HRP a-mouse/a-rabbit IgG; RT; 30 min |
| Amplification | Discovery HQ-HRP; 37°C; 2x 16 min | - |
| DAB development conditions | RT; 5 min | RT; 5 min |
| Wash steps | Reaction buffer | TBST |
| Counterstaining | Hematoxylin II; RT; 16 min | Harris Hematoxylin; RT; 3 min |
| Mounting medium | Glycergel | DPX |
| Autostainer | Discovery Ultra Autostainer | Launch IHC Autostainer |
| Slide scanner | Axioscan.Z1 (Zeiss) | Aperio AT2 slide scanner (Leica Biosystems) |

CC1 buffer: cell conditioning 1 buffer; DAB: 3,3’-diaminobenzidine; DPX: distyrene-plasticizer-xylene-; HRP: horseradish peroxidase; IgG: immunoglobulin G; IHC: immunohistochemistry; RT: room temperature; TBST: Tris-buffered saline with Tween; TF: tissue factor

Supplementary Figures

Figure S1. TF antibody (clone HTF-1) validation by IHC and Western blot analysis.

**A**. IHC of DU145 cells depicting staining with antibody HTF-1; staining was lost upon siRNA knockdown of TF (siTF) but not with scrambled non-specific siRNA (nsCtrl). **B**. A protein band corresponding to the reported molecular weight of full-length TF (45-47 kDa) ^46^ was observed in DU145 cells and to a lesser extent in PC3 cells using Western Blotting. Upon siRNA knockdown of TF, Western Blot staining was lost, confirming the specificity of the antibody. **C**. Brightfield image of the gel to show the MW markers. Vinculin was used a loading control. **D-G.** IHC of cell lines with high (D: A431), low (E: HeLa [ATCC Cat# CCL-2, RRID:CVCL_0030]; F: U-87MG [RRID:CVCL_0022]) and undetectable (G: A-549, RRID:CVCL_0023) TF mRNA expression.

The cellular staining pattern of HTF-1 was similar to that of other anti-TF monoclonal Abs (clones VD8 [AMERICAN DIAGNOSTICA Cat# 4508, RRID:AB_400658] and IIID8 [AMERICAN DIAGNOSTICA Cat# 4509, RRID:AB_400659]) (data not shown). TF protein expression as determined by flow cytometry and Western Blot correlated with mRNA expression as described in the Cancer Cell Line Encyclopedia (CCLE, <https://www.broadinstitute.org/ccle>, RRID:SCR_013836) from the Broad Institute.


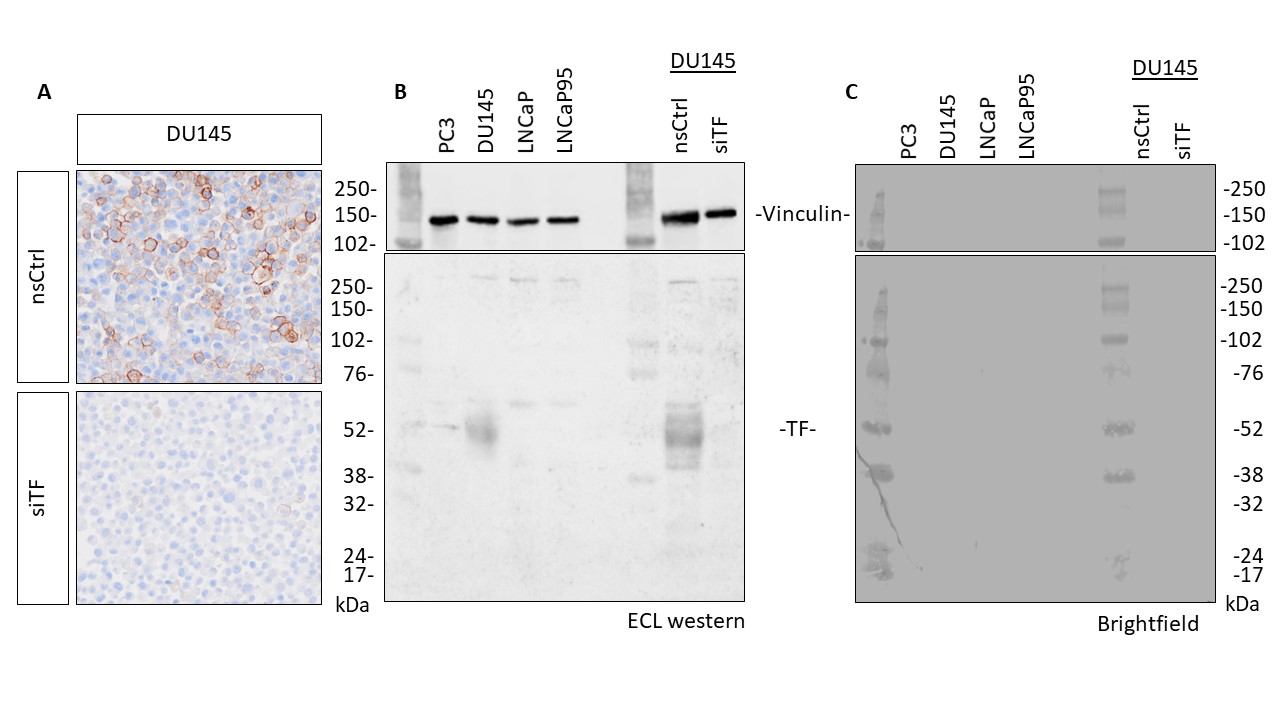


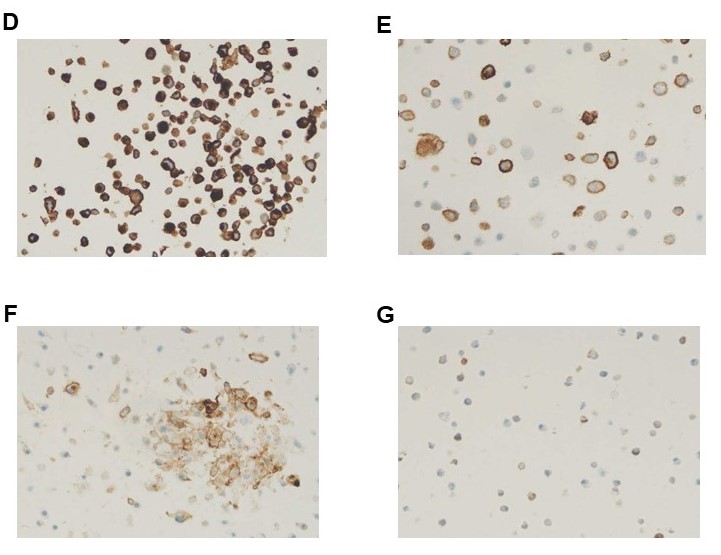


Figure S2. Staining of cell lines with known TF expression demonstrates that the IHC methods are reproducible and have a broad assay dynamic range

**A.** Two different IHC protocols were compared using tumor cell lines with known low (PC-3), moderate (786-0, HCT 116) and high (BxPC-3) TF expression. Tumor cell lines were stained with mouse anti-TF antibody (clone HTF-1; brown) and nuclei were counterstained with hematoxylin (blue; original magnification 20x; scale bars: 100 mm). Pictures from one representative run of three independent experiments are shown. **B.** TF H-scores for the different tumor cell lines were calculated for all three experiments according to **Equation 1** (main manuscript) and depicted as mean ± SD. TF H-scores ranged from low in PC-3 cells (TF H-score: 0-10), to moderate in 786-0 cells (TF H-score: 175-180) and HCT 116 cells (TF H-score: 180-200), to high in BxPC-3 cells (TF H-score: 270-300) and were comparable between IHC method 1 and 2.

**
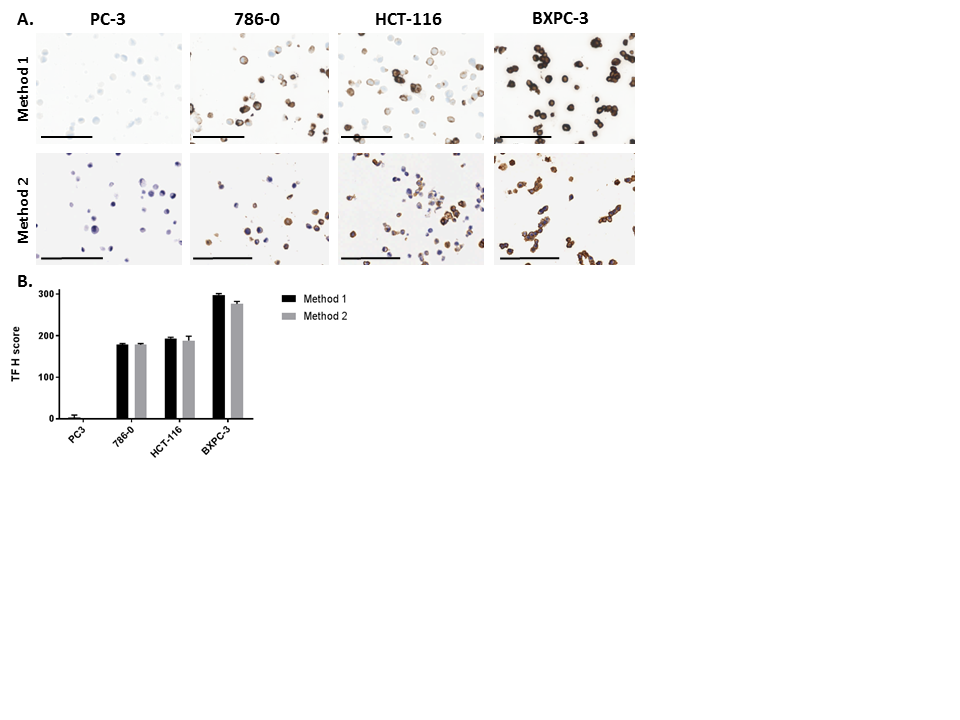
**

Figure S3. Expression of TF antigen in FFPE tissue is stable over time

Normal kidney tissue was formalin-fixed and paraffin-embedded directly after excision. At several time intervals after fixation (0, 3.5, 6.5, 10 months), sections were freshly cut and stained with mouse anti-human TF (clone HTF-1; 2.5 µg/mL). Pictures show representative examples of TF staining (brown) and nuclei (blue) of a renal corpuscle in the kidney biopsy, which stained with an equal intensity at all investigated time intervals (original magnification 20x; scale bars: 50 mm).


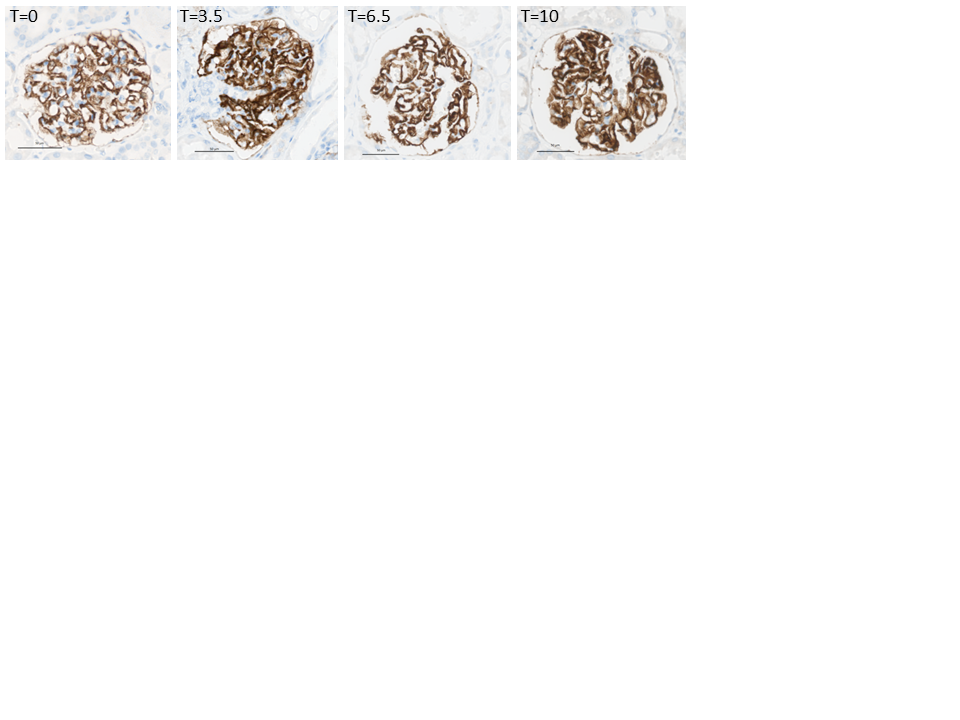


Figure S4. TF H-scores in patient-matched biopsies stratified by disease stage

TF staining (mouse anti-human TF [clone HTF-1; 3 µg/mL; brown]; nuclei were counterstained with hematoxylin [blue]). was performed on patient-matched T1 and T2 biopsies of patients that progressed from an early to an advanced stage of disease between T1 and T2: cervical (n=5), ovarian (n=7), prostate (n=10), endometrial (n=4) and bladder (n=1; in graph ‘other cancer types’) cancer, and on matched T1 and T2 tumor biopsies of patients that remained in the same disease stage between T1 and T2: cervical (n=6), ovarian (n=19), prostate (n=16), endometrial (n=4), esophageal (n=3; in graph ‘other cancer types’), lung (n=2; in graph ‘other cancer types’) and bladder (n=1; in graph ‘other cancer types’). TF H-scores were calculated according to **Equation 1**. Statistical analysis was performed using a Wilcoxon signed rank test and indicated no significant differences in TF H-scores between T1 and T2 in all analyzed groups. Data shown are H-scores per patient at T1 and T2. Colors indicate the disease state at T1 and T2 (E: early; A: advanced).

Supplementary References

1. Guan M, Jin J, Su B, Liu WW, Lu Y. Tissue factor expression and angiogenesis in human glioma. *Clin Biochem* 2002;35: 321-5.

2. Hamada K, Kuratsu J, Saitoh Y, Takeshima H, Nishi T, Ushio Y. Expression of tissue factor correlates with grade of malignancy in human glioma. *Cancer* 1996;77: 1877-83.

3. Takano S, Tsuboi K, Tomono Y, Mitsui Y, Nose T. Tissue factor, osteopontin, alphavbeta3 integrin expression in microvasculature of gliomas associated with vascular endothelial growth factor expression. *Br J Cancer* 2000;82: 1967-73.

4. Takeshima H, Nishi T, Kuratsu J, Kamikubo Y, Kochi M, Ushio Y. Suppression of the tissue factor-dependent coagulation cascade: a contributing factor for the development of intratumoral hemorrhage in glioblastoma. *Int J Mol Med* 2000;6: 271-6.

5. Chen K, Li Z, Jiang P, Zhang X, Zhang Y, Jiang Y, He Y, Li X. Co-expression of CD133, CD44v6 and human tissue factor is associated with metastasis and poor prognosis in pancreatic carcinoma. *Oncol Rep* 2014;32: 755-63.

6. Haas SL, Jesnowski R, Steiner M, Hummel F, Ringel J, Burstein C, Nizze H, Liebe S, Lohr JM. Expression of tissue factor in pancreatic adenocarcinoma is associated with activation of coagulation. *World J Gastroenterol* 2006;12: 4843-9.

7. Khorana AA, Ahrendt SA, Ryan CK, Francis CW, Hruban RH, Hu YC, Hostetter G, Harvey J, Taubman MB. Tissue factor expression, angiogenesis, and thrombosis in pancreatic cancer. *Clin Cancer Res* 2007;13: 2870-5.

8. Nitori N, Ino Y, Nakanishi Y, Yamada T, Honda K, Yanagihara K, Kosuge T, Kanai Y, Kitajima M, Hirohashi S. Prognostic significance of tissue factor in pancreatic ductal adenocarcinoma. *Clin Cancer Res* 2005;11: 2531-9.

9. Wojtukiewicz MZ, Rucinska M, Zacharski LR, Kozlowski L, Zimnoch L, Piotrowski Z, Kudryk BJ, Kisiel W. Localization of blood coagulation factors in situ in pancreatic carcinoma. *Thromb Haemost* 2001;86: 1416-20.

10. Cocco E, Varughese J, Buza N, Bellone S, Glasgow M, Bellone M, Todeschini P, Carrara L, Silasi DA, Azodi M, Schwartz PE, Rutherford TJ, et al. Expression of tissue factor in adenocarcinoma and squamous cell carcinoma of the uterine cervix: implications for immunotherapy with hI-con1, a factor VII-IgGFc chimeric protein targeting tissue factor. *BMC Cancer* 2011;11: 263.

11. Srinivasan R, Bogdanov VY. Alternatively spliced tissue factor: discovery, insights, clinical implications. *Front Biosci (Landmark Ed)* 2011;16: 3061-71.

12. Zhao X, Cheng C, Gou J, Yi T, Qian Y, Du X, Zhao X. Expression of tissue factor in human cervical carcinoma tissue. *Exp Ther Med* 2018;16: 4075-81.

13. Goldin-Lang P, Tran QV, Fichtner I, Eisenreich A, Antoniak S, Schulze K, Coupland SE, Poller W, Schultheiss HP, Rauch U. Tissue factor expression pattern in human non-small cell lung cancer tissues indicate increased blood thrombogenicity and tumor metastasis. *Oncol Rep* 2008;20: 123-8.

14. Koomagi R, Volm M. Tissue-factor expression in human non-small-cell lung carcinoma measured by immunohistochemistry: correlation between tissue factor and angiogenesis. *Int J Cancer* 1998;79: 19-22.

15. Regina S, Rollin J, Blechet C, Iochmann S, Reverdiau P, Gruel Y. Tissue factor expression in non-small cell lung cancer: relationship with vascular endothelial growth factor expression, microvascular density, and K-ras mutation. *J Thorac Oncol* 2008;3: 689-97.

16. Sawada M, Miyake S, Ohdama S, Matsubara O, Masuda S, Yakumaru K, Yoshizawa Y. Expression of tissue factor in non-small-cell lung cancers and its relationship to metastasis. *Br J Cancer* 1999;79: 472-7.

17. Wang B, Berger M, Masters G, Albone E, Yang Q, Sheedy J, Kirksey Y, Grimm L, Wang B, Singleton J, Soltis D. Radiotherapy of human xenograft NSCLC tumors in nude mice with a 90Y-labeled anti-tissue factor antibody. *Cancer Biother Radiopharm* 2005;20: 300-9.

18. Kaido T, Oe H, Yoshikawa A, Mori A, Arii S, Imamura M. Tissue factor is a useful prognostic factor of recurrence in hepatocellular carcinoma in 5-year survivors. *Hepatogastroenterology* 2005;52: 1383-7.

19. Poon RT, Lau CP, Ho JW, Yu WC, Fan ST, Wong J. Tissue factor expression correlates with tumor angiogenesis and invasiveness in human hepatocellular carcinoma. *Clin Cancer Res* 2003;9: 5339-45.

20. Cocco E, Hu Z, Richter CE, Bellone S, Casagrande F, Bellone M, Todeschini P, Krikun G, Silasi DA, Azodi M, Schwartz PE, Rutherford TJ, et al. hI-con1, a factor VII-IgGFc chimeric protein targeting tissue factor for immunotherapy of uterine serous papillary carcinoma. *Br J Cancer* 2010;103: 812-9.

21. Fadare O, Renshaw IL, Liang SX. Expression of tissue factor and heparanase in endometrial clear cell carcinoma: possible role for tissue factor in thromboembolic events. *Int J Gynecol Pathol* 2011;30: 252-61.

22. Abdulkadir SA, Carvalhal GF, Kaleem Z, Kisiel W, Humphrey PA, Catalona WJ, Milbrandt J. Tissue factor expression and angiogenesis in human prostate carcinoma. *Hum Pathol* 2000;31: 443-7.

23. Akashi T, Furuya Y, Ohta S, Fuse H. Tissue factor expression and prognosis in patients with metastatic prostate cancer. *Urology* 2003;62: 1078-82.

24. Kaushal V, Mukunyadzi P, Siegel ER, Dennis RA, Johnson DE, Kohli M. Expression of tissue factor in prostate cancer correlates with malignant phenotype. *Appl Immunohistochem Mol Morphol* 2008;16: 1-6.

25. Ohta S, Wada H, Nakazaki T, Maeda Y, Nobori T, Shiku H, Nakamura S, Nagakawa O, Furuya Y, Fuse H. Expression of tissue factor is associated with clinical features and angiogenesis in prostate cancer. *Anticancer Res* 2002;22: 2991-6.

26. Yao JL, Ryan CK, Francis CW, Kohli M, Taubman MB, Khorana AA. Tissue factor and VEGF expression in prostate carcinoma: a tissue microarray study. *Cancer Invest* 2009;27: 430-4.

27. Chen L, Luo G, Tan Y, Wei J, Wu C, Zheng L, Zhang X, Xu N. Immunolocalisation of tissue factor in esophageal cancer is correlated with intratumoral angiogenesis and prognosis of the patient. *Acta Histochem* 2010;112: 233-9.

28. Motoori M, Yano M, Tomita Y, Takahashi H, Tanaka K, Sugimura K, Kishi K, Fujiwara Y, Miyoshi N, Akita H, Goto K, Marubashi S, et al. Tissue factor predicts response to chemotherapy in esophageal cancer. *J Surg Res* 2014;191: 99-105.

29. Abu Saadeh F, Norris L, O'Toole S, Mohamed BM, Langhe R, O'Leary J, Gleeson N. Tumour expresion of tissue factor and tissue factor pathway inhibitor in ovarian cancer- relationship with venous thrombosis risk. *Thromb Res* 2013;132: 627-34.

30. Cocco E, Varughese J, Buza N, Bellone S, Lin KY, Bellone M, Todeschini P, Silasi DA, Azodi M, Schwartz PE, Rutherford TJ, Carrara L, et al. Tissue factor expression in ovarian cancer: implications for immunotherapy with hI-con1, a factor VII-IgGF(c) chimeric protein targeting tissue factor. *Clin Exp Metastasis* 2011;28: 689-700.

31. Uno K, Homma S, Satoh T, Nakanishi K, Abe D, Matsumoto K, Oki A, Tsunoda H, Yamaguchi I, Nagasawa T, Yoshikawa H, Aonuma K. Tissue factor expression as a possible determinant of thromboembolism in ovarian cancer. *Br J Cancer* 2007;96: 290-5.

32. Yokota N, Koizume S, Miyagi E, Hirahara F, Nakamura Y, Kikuchi K, Ruf W, Sakuma Y, Tsuchiya E, Miyagi Y. Self-production of tissue factor-coagulation factor VII complex by ovarian cancer cells. *Br J Cancer* 2009;101: 2023-9.

33. Patry G, Hovington H, Larue H, Harel F, Fradet Y, Lacombe L. Tissue factor expression correlates with disease-specific survival in patients with node-negative muscle-invasive bladder cancer. *Int J Cancer* 2008;122: 1592-7.

34. Kocaturk B, Tieken C, Vreeken D, Unlu B, Engels CC, de Kruijf EM, Kuppen PJ, Reitsma PH, Bogdanov VY, Versteeg HH. Alternatively spliced tissue factor synergizes with the estrogen receptor pathway in promoting breast cancer progression. *J Thromb Haemost* 2015;13: 1683-93.

35. Kocaturk B, Van den Berg YW, Tieken C, Mieog JS, de Kruijf EM, Engels CC, van der Ent MA, Kuppen PJ, Van de Velde CJ, Ruf W, Reitsma PH, Osanto S, et al. Alternatively spliced tissue factor promotes breast cancer growth in a beta1 integrin-dependent manner. *Proc Natl Acad Sci U S A* 2013;110: 11517-22.

36. Ryden L, Grabau D, Schaffner F, Jonsson PE, Ruf W, Belting M. Evidence for tissue factor phosphorylation and its correlation with protease-activated receptor expression and the prognosis of primary breast cancer. *Int J Cancer* 2010;126: 2330-40.

37. Stampfli SF, Akhmedov A, Hausladen S, Varga Z, Dedes KJ, Hellermann J, Luscher TF, Kristiansen G, Tanner FC, Breitenstein A. Tissue Factor Expression Does Not Predict Mortality in Breast Cancer Patients. *Anticancer Res* 2017;37: 3259-64.

38. Sturm U, Luther T, Albrecht S, Flossel C, Grossmann H, Muller M. Immunohistological detection of tissue factor in normal and abnormal human mammary glands using monoclonal antibodies. *Virchows Arch A Pathol Anat Histopathol* 1992;421: 79-86.

39. Ueno T, Toi M, Koike M, Nakamura S, Tominaga T. Tissue factor expression in breast cancer tissues: its correlation with prognosis and plasma concentration. *Br J Cancer* 2000;83: 164-70.

40. Vrana JA, Stang MT, Grande JP, Getz MJ. Expression of tissue factor in tumor stroma correlates with progression to invasive human breast cancer: paracrine regulation by carcinoma cell-derived members of the transforming growth factor beta family. *Cancer Res* 1996;56: 5063-70.

41. Wojtukiewicz MZ, Sierko E, Skalij P, Kaminska M, Zimnoch L, Brekken RA, Thorpe PE. Granulocyte-Colony Stimulating Factor Receptor, Tissue Factor, and VEGF-R Bound VEGF in Human Breast Cancer In Loco. *Adv Clin Exp Med* 2016;25: 505-11.

42. Nakasaki T, Wada H, Shigemori C, Miki C, Gabazza EC, Nobori T, Nakamura S, Shiku H. Expression of tissue factor and vascular endothelial growth factor is associated with angiogenesis in colorectal cancer. *Am J Hematol* 2002;69: 247-54.

43. Seto S, Onodera H, Kaido T, Yoshikawa A, Ishigami S, Arii S, Imamura M. Tissue factor expression in human colorectal carcinoma: correlation with hepatic metastasis and impact on prognosis. *Cancer* 2000;88: 295-301.

44. Shigemori C, Wada H, Matsumoto K, Shiku H, Nakamura S, Suzuki H. Tissue factor expression and metastatic potential of colorectal cancer. *Thromb Haemost* 1998;80: 894-8.

45. Wan Y, Wu N, Wang Z, Ju X, Zhu J, Liu Y, Tang J, Huang Y. Relationship between tissue factor expression and hepatic metastasis and prognosis in rectal cancer. *Zhonghua Zhong Liu Za Zhi* 2002;24: 378-80.

46. Bach RR. Initiation of coagulation by tissue factor. CRC Crit Rev Biochem 1988;23: 339-68.
